# Comparative Study of Surface-Coated MoS_2_ on the Multiscale Tribological Performance of Cu-Based Composites

**DOI:** 10.3390/ma19061123

**Published:** 2026-03-13

**Authors:** Yueqi Li, Qi Li, Haibin Zhou, Xuan He, Boxian Li, Wenhan Liu, Yuxuan Xu, Taimin Gong, Minwen Deng, Xiubo Liu, Pingping Yao, Qiangguo Chen

**Affiliations:** 1 Hunan Province Key Laboratory of Materials Surface/Interface Science & Technology, Central South University of Forestry & Technology, Changsha 410004, China20231100257@csuft.edu.cn (Q.L.); 15897294467@163.com (W.L.);; 2 State Key Laboratory of Powder Metallurgy, Central South University, Changsha 410083, China; 3 School of Chemistry, Chemical Engineering and Life Sciences, Wuhan University of Technology, Wuhan 430000, China

**Keywords:** Cu-based composite, interface, tribology performance, wear mechanism, lubricating component

## Abstract

**Highlights:**

- The Ni coating suppresses MoS_2_ decomposition during sintering.
- MoS_2_@Ni forms a diffusion-bonded interface with higher load-bearing capacity.
- Cu coating promotes interfacial reaction and defect formation.
- Cu-BC-MoS_2_@Ni exhibits lower wear rate under high braking energy density.

**Abstract:**

MoS_2_ acts as a high-performance lubricant, enhancing friction material stability, reducing wear and noise under extreme conditions, and preserving friction pair performance. However, its tendency to decompose and poor matrix wettability make surface modification essential for effective use in Cu-based composites. In this study, comprehensive investigations combining macro-scale and micro-scale friction experiments were conducted to examine the interfacial friction behavior of MoS_2_ with different coatings and its tribological effects on copper-based composites under varying braking energy densities. The results indicate that the nickel coating suppressed MoS_2_ decomposition, forming a high-strength diffusion interface with the matrix. This enhances the frictional stability and suppresses interfacial defect formation during micro-friction tests. However, the copper coating formed a poor-strength diffusion-reacting interface with matrix, leading to unstable friction at the interface and interface failure. Coating-dependent interfacial properties and micro-friction behaviors lead to varying tribological performance in Cu-based composites with MoS_2_ during macro-friction tests. Nickel-plated MoS_2_ (MoS_2_@Ni) exhibits superior lubrication and frictional stability. The friction coefficients of Cu-based composites with MoS_2_@Ni under low, medium and high working conditions are 0.36, 0.3 and 0.24, respectively, which are 6%, 12% and 13% lower than those of copper-plated MoS_2_ (MoS_2_@Cu). Meanwhile, its friction stability is 0.8, 0.6 and 0.58, respectively. With rising braking energy density, wear in Cu-based composites transitions from ploughing to oxidation and then to delamination. Defective MoS_2_@Cu/matrix interfaces intensify delamination wear caused by the unstable fracture of subsurface plastic deformation layer cracks at higher energy density.

## 1. Introduction

Cu-based composites are extensively used in braking applications due to their desirable combination of stable friction coefficient, excellent wear resistance, good thermal stability, and high mechanical strength [1,2,3,4,5,6,7]. However, the increasing demands of modern high-speed and heavy-duty braking systems require further enhancement of their tribological performance under extreme operating conditions [8,9,10,11]. Consequently, the existing friction materials struggle to meet the demands of braking systems for stability, wear resistance, and high-temperature tolerance—particularly under complex conditions involving high speeds, heavy loads, and high braking energy densities. Therefore, there is an urgent need in material science to develop Cu-based composite with enhanced performance that can operate stably under extreme conditions for extended periods [12,13,14].

The optimized design of materials constitutes a crucial approach to enhancing the performance of Cu-based composites. A key strategy for optimizing these composites involves the incorporation of functional additives, such as reinforcing and lubricating phases, to tailor their mechanical and tribological properties [15,16,17,18]. For instance, researchers, like Ashish Saurabhet et al. [18], have found that the application of MoS_2_ nanosheets as solid lubricants in automotive brake disc system friction materials can result in excellent friction platform coverage, effectively reducing wear and minimizing wear on the reverse disc. Additionally, Panahsadat Fasihi et al. [19] examined the influence of solid lubricants, specifically graphite and molybdenum disulfide, applied at the wheel–rail interface on the wear mechanisms and the surface topography of hypereutectoid rail steel. Among all components, lubricating phases are particularly important for developing high-performance Cu-based composites. They smooth the engagement process and optimize thermal distribution during braking, making these composites suitable for high-speed and heavy-duty applications [20,21,22,23].

MoS_2_, which exhibits superior lubrication performance across diverse environmental conditions, is a high-performance and widely utilized critical lubricating component, serving as a highly effective friction stabilizer. However, its poor wettability and weak interfacial stability with the Cu matrix lead to a reduction in its lubrication effectiveness [24,25,26,27]. Consequently, in most current Cu-based friction material formulations, MoS_2_ is only added in small quantities to supplement the lubrication provided by the primary solid lubricant, graphite [28,29,30,31,32]. To improve the stability and interfacial compatibility of MoS_2_, extensive attention has been directed towards research concerning surface modification treatments of MoS_2_. Tian-xu Qiu et al. [23] studied the frictional properties of nickel-plated MoS_2_ in Cu-based composites. They found that nickel plating enhances the adhesion between MoS_2_ and the substrate, improving the material’s strength and hardness. Wang Hui-ling et al. [33] introduced copper-plated MoS_2_ into a Cu-based composite. The results indicated that the copper coating modified the original mechanical bonding interface between the matrix and the MoS_2_, leading to a notable improvement in both strength and wear resistance. Moreover, although the efficacy of various coating treatments in enhancing MoS_2_ properties has been demonstrated, this stands in contrast to the current absence of a thorough comparative analysis across different coating types. However, previous studies have not systematically examined the correlations among interface performance, micro-friction characteristics of lubricating components/interfaces, and macro-scale friction behavior of a Cu-based composite/counterpart system [29]. Çiçek B. et al. [34] demonstrated that TiNi/MoS_2_ functional coatings deposited on bearing steel exhibited excellent tribological performance, with friction coefficients as low as 0.1 under both ambient and high-temperature conditions. Wang G. et al. [35] revealed that the friction behavior of MoS_2_ coatings on copper is temperature-dependent: the coating maintains low friction up to ~200 °C, above which oxidation and structural degradation cause significant increases in friction and wear.

This study systematically investigates the effects of Ni and Cu coatings on MoS_2_ on the interfacial properties and multiscale tribological behavior of Cu-based composites. The influence of coated MoS_2_ on wear mechanisms under varying braking energy densities is also elucidated, aiming to guide the design of high-performance braking materials.

## 2. Experimental Section

### 2.1. Material Preparation

The raw powders utilized in this study are presented below: electrolytic copper powder serves as the matrix component, and iron (Fe) and ferrochrome are employed for alloy strengthening and friction enhancement (alloy component and friction component). To prevent abnormal wear that could compromise test data reliability, CrFe was added as a reinforcing component to the Cu-based composites containing MoS_2_@Cu and MoS_2_@Ni. This addition helps regulate friction performance and ensures smooth braking (the MoS_2_ utilized in this study includes Cu-coated MoS_2_ and Ni-coated MoS_2_, hereafter referred to as MoS_2_@Cu and MoS_2_@Ni, respectively) to regulate friction performance and ensure a smooth braking process. Additionally, MoS_2_ with various coatings and graphite are incorporated as lubrication components. The detailed technical parameters are shown in Table 1.

MoS_2_@Cu and MoS_2_@Ni powders were synthesized by electroless plating (chemical reduction plating). For the electroless copper plating, the bath composition consisted of CuSO_4_·5H_2_O (15 g/L) and EDTA-2Na (30 g/L) as complexing agents, NaOH (adjusted to pH 12–13) and formaldehyde (10 mL/L) as reducing agents, and α,α’-bipyridine as stabilizer. The plating temperature was maintained at 60 ± 2 °C with continuous stirring for 40 min.

For the electroless nickel plating, the bath contained NiSO_4_·6H_2_O (25 g/L), NiCl_2_·6H_2_O (10 g/L), NaH_2_PO_2_ (20 g/L) as a reducing agent, and sodium borohydride as an auxiliary reducing agent. The pH value was adjusted to 9–10 and the temperature was controlled at 85 ± 2 °C.

The powder mixtures were prepared according to the proportions specified in Table 2. Samples 1# (Cu-MoS_2_@Ni) and 2# (Cu-MoS_2_@Cu) were designed with an ultra-simplified formulation to isolate the effects of other components. They were used to test the stability of differently coated MoS_2_, the characteristics of the interfaces formed between MoS_2_@Ni/MoS_2_@Cu and the copper matrix, the micro-tribological behavior, and the influence of the coatings on the physical and mechanical properties of the composites. In contrast, Samples 3# (Cu-BC-MoS_2_@Cu) and 4# (Cu-BC-MoS_2_@Ni) were formulated based on our research group’s previously developed composition for high-speed train brake materials. The objective was to conduct a comprehensive assessment of the impact of two types of MoS_2_ on the macro-tribological performance under diverse braking energy density conditions.

**Figure 1:**
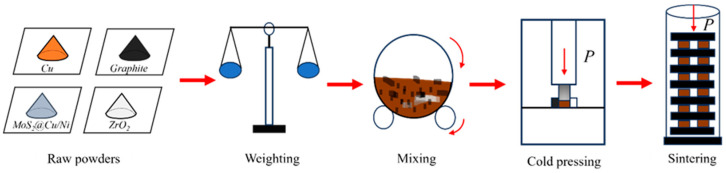
Processing steps of Cu-based friction materials.

Figure 1 illustrates the fabrication process, which comprised the following four steps: (1) weighing and batching of the raw material powders, including molybdenum disulfide MoS_2_. (2) Homogeneous mixing of the batched powders for 7 h using a V-type mixer (Wuxi Chenli powder equipment Co., Ltd., Wuxi, China). (3) Compacting the mixed powders in a cold-pressing die at 380–530 MPa to form green compacts with sufficient strength. (4) Pressure-assisted sintering of the green compacts to promote metallurgical bonding both among the matrix constituents and between the copper-/nickel-coated MoS_2_ and the matrix. The final powder metallurgy test materials were prepared in a customized sintering furnace under a hydrogen protective atmosphere, with a sintering pressure of 2.8 MPa and a dwell time of 2.5 h at 870 °C. Following sintering, the specimens were sectioned, mounted, ground, and polished to obtain mirror-smooth, scratch-free metallographic samples.

### 2.2. Experimental Method

The microstructure of the copper-based composites was characterized using a metallurgical microscope (Leica-Q550 SiChangYue Company, Shanghai, China). The phases present in the samples were analyzed by X-ray diffraction (XRD, Rigaku RapidIIR, Rigaku Corporation, Tokyo, Japan). XRD measurements were conducted using Cu Kα radiation (λ = 1.5406 Å) at 40 kV and 40 mA. The scanning range was 10–80° (2θ) with a step size of 0.02° and a scanning speed of 5°/min. Detailed interfacial features, as well as the wear surfaces, subsurfaces, and wear debris, were examined using a scanning electron microscope (SEM) equipped with energy-dispersive spectroscopy (EDS), sourced from Quanta FEG 250 (Thermo Fisher Scientific, Waltham, MA, USA). SEM images were obtained using secondary electron (SE) mode for surface morphology observation, while backscattered electron (BSE) mode was employed to distinguish phase contrast at the interfaces.

The apparent hardness of the sample was determined according to the Brinell hardness test method, using a 310HBW-3000 digital Brinell hardness tester (Huayin Company, Laizhou, China). During the testing process, a hard alloy ball with a diameter of 2.5 mm was selected as the indenter, and a test force was applied to the specimen at room temperature. The test force was set to 62.5 kgf (612.5 N), and the test force was maintained for 30 s. To reduce measurement errors and ensure data representativeness, four different positions were selected on the surface of each sample as test points, and the arithmetic mean was taken as the final apparent hardness value of the sample.

**Figure 2:**
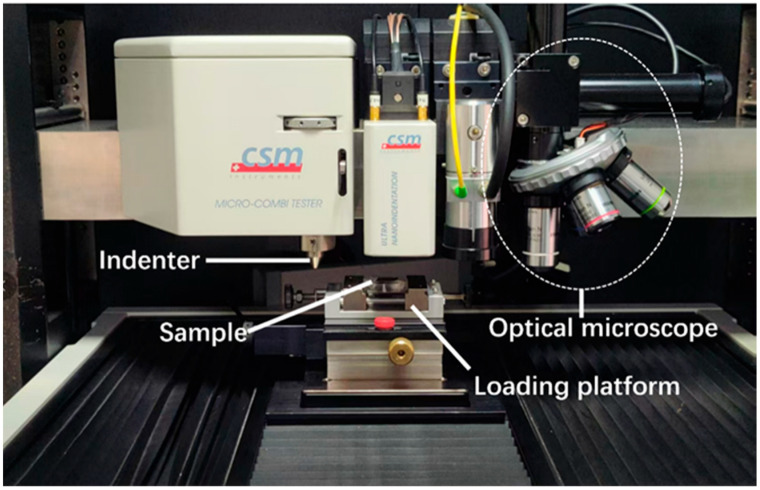
Micro-friction testing machine.

The purpose of micro-friction testing is the elucidation of the inherent tribological characteristics of the composite’s constituents and the interfacial tribological response between them and the matrix. These tests were conducted using a CSM Micro-Combi Tester (SiChangYue Company, Shanghai, China) as shown in Figure 2. A micro-friction testing setup was used for interface scratch experiments. A Rockwell diamond indenter (R = 100 μm) slid across the sample surface at 0.2 mm/min under normal loads of 0.05 N, 0.075 N, and 0.15 N. Tangential force (Ft), normal force (Fn), and penetration depth (Pd) were continuously recorded.

The micro-friction testing procedure comprised the following steps: (1) Pre-scan phase: Prior to the commencement of the friction test, the indenter was traversed across the target area under an ultra-low normal load to acquire the surface topography. (2) Test execution phase: The indenter was then retracted to its initial position and subsequently slid over the designated test area at a constant velocity of 0.1 mm/min under a preset normal load, completing the predefined sliding distance. (3) Post-scan phase: Upon completion of the friction test, the indenter was returned to the starting point and the worn surface morphology was recorded under an ultra-low normal load for comparative analysis. Throughout the entire testing procedure, the tangential force (Ft), normal force (Fn), and penetration depth (Pd) were continuously monitored and recorded. The specific parameters for the micro-friction tests are listed in Table 3.

**Figure 3:**
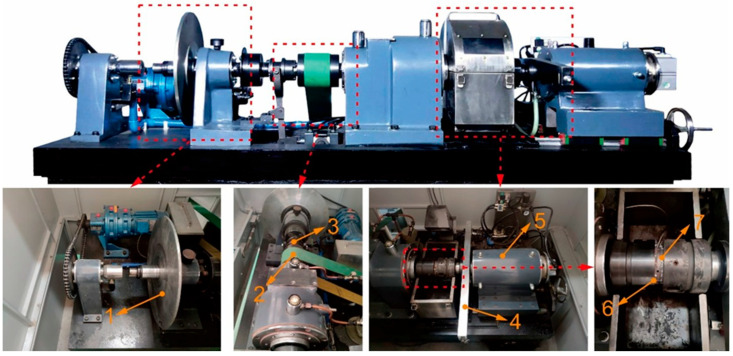
Schematic diagram of the MM3000 friction tester used for macro-scale braking experiments. (1) Inertia component; (2) transmission belt; (3) clutch; (4) torque sensor; (5) hydraulic cylinder; (6,7) friction material specimen and counterpart disc.

This study employed an MM3000 tester (Xi’an Shunyi Electronics Company, Xi’an, China) (Figure 3) to assess the effect of MoS_2_ with different coatings on the tribological performance of Cu-based composites across a range of braking energy densities. The counterpart material was 30CrMnSiA alloy steel. For testing, all materials were fabricated into annular specimens (75/53 mm OD/ID) and ground to achieve a surface finish of Ra = 0.6 µm.

The macroscopic scale experiments were conducted using an MM-3000 friction testing machine. The inertia setting of the experiment was 1.035 kg·m^2^, and the energy densities tested were 30, 50, and 100 J/mm^2^, respectively. A constant test pressure of 0.6 MPa was applied. Under each test condition, the experiment was repeated ten times. The specimen rings were precision-machined to have an outer diameter of 37.5 mm and an inner diameter of 26.5 mm. The counter material used was 30CrMnSiVA alloy steel with a hardness of HB 39–42 [4].

The macroscopic friction test consists of the following steps: (1) before the experiment, assemble the friction test ring and the friction pair, and set the experimental parameters. (2) During the friction experiment, the MM3000 begins to rotate. Once the set parameters are reached, pressure is applied, and friction occurs between the test ring and the friction pair until the shaft stops rotating. At this point, the sensor collects the friction data. (3) After the experiment, when the shaft stops, wait for the test ring and friction pair to cool down to room temperature before starting the next test. During each experiment, the sensor collects instantaneous friction data, including the instantaneous friction coefficient, steady-state coefficient, and other relevant parameters. The parameters for the macro-friction tests are detailed in Table 4. The following formula calculates the instantaneous friction coefficient in friction experiments [36]:

(1) $$\;\mu_{s}=\frac{M_{s}}{RF_{n}}\;$$

In the equation, $\mu_{s}$ represents the instantaneous friction coefficient, Ms denotes the torque collected by the torque sensor, R stands for the effective radius of the test ring, and Fn represents the applied load pressure.

During the experimental process, the average friction coefficient is calculated as follows:

(2) $$\mu_{a}=\int_{0}^{t}\mu dt\;$$

In the equation, t represents the duration of the friction process, and μa denotes the average friction coefficient. Data recording begins at 95% of the pressure loading and continues until the predetermined pressure is reached.

The stability coefficient ω during the friction process is calculated as follows [37,38]:

(3) $$\omega =\frac{\mu_{a}}{\mu_{max}}\;$$

During the friction assessment described by the equation, μmax denotes the maximum value of the friction coefficient.

The wear volume during the experimental process is the volume wear per unit energy, as shown in the following equation:

(4) $$W=\frac{{(l}_{t}-l_{i})S\rho }{N\cdot E}\;$$

In the equation, $l_{t}$ and $l_{s}$ represent the linear wear of the friction material or counterpart material before and after repeated testing, respectively. S denotes the nominal contact area of the friction pair, ρ stands for the density of the friction material, N indicates the number of tests during the friction process, E represents the energy endured by the material and ω is the rotational angular velocity, which can be calculated using the following formula:

(5) $$E=\frac{1}{2}I\omega^{2}$$

## 3. Results

### 3.1. The Morphology and Mechanical Properties of Modified MoS_2_ Powder and Its Interface Formation with the Matrix

**Figure 4:**
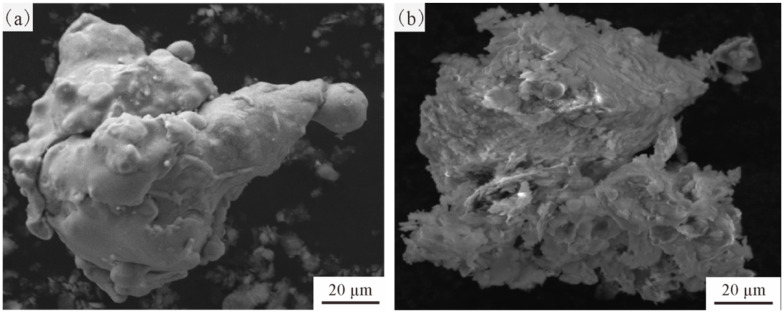
Typical morphology of MoS_2_@Cu and MoS_2_@Ni particles (BSE): (**a**) MoS_2_@Cu; (**b**) MoS_2_@Ni.

The application of Cu and Ni coatings on MoS_2_ alters the particle morphology compared to uncoated MoS_2_. Consequently, prior to experimentation, a comprehensive and in-depth analysis of the characteristics of MoS_2_ powders with different coatings is essential. Figure 4 illustrates the typical morphological features of MoS_2_ with different coatings. It can be observed from the figure that both types of MoS_2_ particles exhibit complex irregular shapes. The surfaces of the MoS_2_ particles (MoS_2_@Cu and MoS_2_@Ni), however, were fully covered by a relatively dense metallic coating. The observed particle morphology is a direct consequence of the preparation process used. MoS_2_ is mostly fine powder prepared by chemical methods. To achieve a specific particle size, granulation is often employed to agglomerate the fine powder, resulting in larger particles of a uniform size. Therefore, the particles usually show complex shapes. Typically, agglomerated MoS_2_ particles possess a rougher surface and a higher specific surface area. This characteristic is beneficial for the deposition of a metal coating during the subsequent coating process, which in turn facilitates the formation of MoS_2_ particles with a smooth and continuous metallic layer. Consequently, this enhances the bonding strength between MoS_2_ and the matrix.

**Figure 5:**
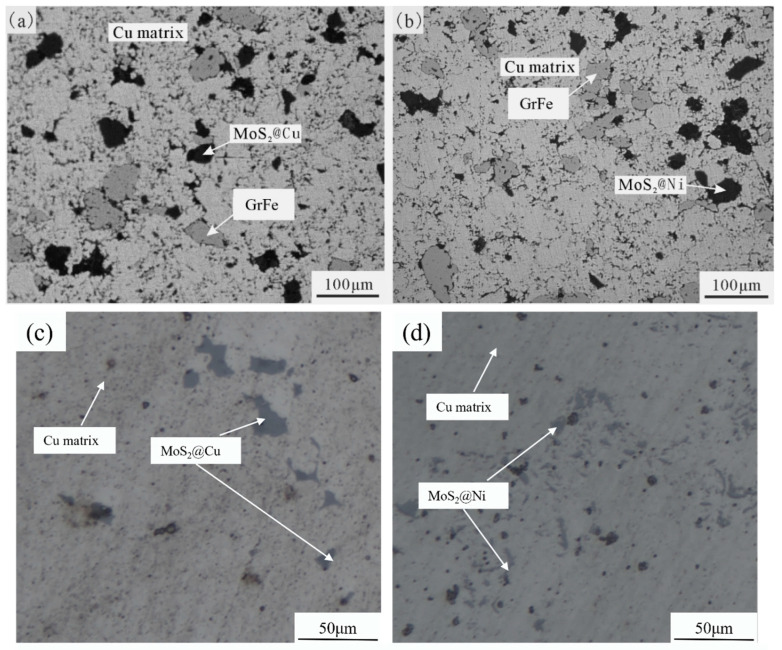
The optical microstructure photos of Cu-based composites: (**a**) Cu-BC-MoS_2_@Cu (3#); (**b**) Cu-BC-MoS_2_@Ni (4#); (**c**) Cu-MoS_2_@Cu (1#); (**d**) Cu-MoS_2_@Ni (2#).

As shown in Figure 5a, the black phase corresponds to MoS_2_-Cu, which appears to be well-integrated within the Cu matrix at this magnification. Figure 5b illustrates the microstructure of MoS_2_@Ni, showing that MoS_2_@Ni also forms a stable interface with the matrix. Figure 5c,d show the microstructures of Cu-MoS_2_@Cu and Cu-MoS_2_@Ni, respectively. The modified MoS_2_ particles are dispersed throughout the Cu matrix, although some degree of agglomeration and local variation in particle density can be observed, which is typical for powder metallurgy processed composites.

**Figure 6:**
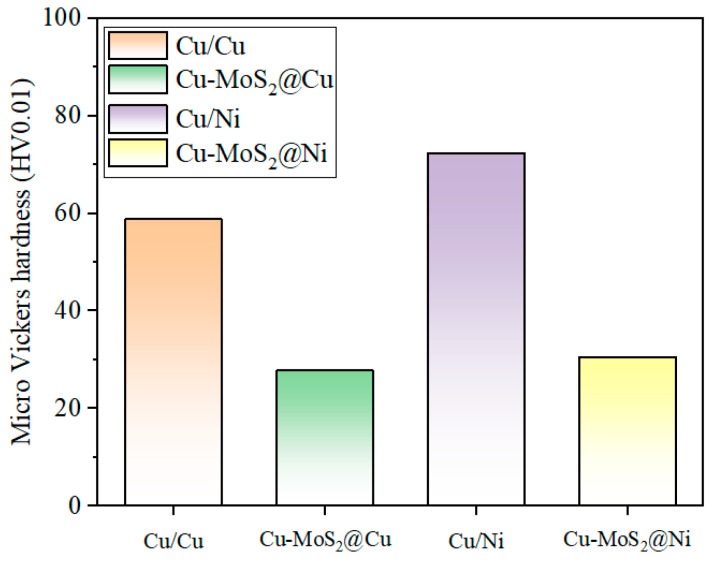
Micro-Vickers hardness for Cu-MoS_2_@Cu, Cu-MoS_2_@Ni and Cu matrix.

Figure 6 presents the microhardness values of the Cu-MoS_2_@Cu and Cu-MoS_2_@Ni samples, as well as their respective Cu matrix. The two materials exhibited relatively low and comparable microhardness. It is noteworthy that the copper matrix in the Cu-MoS_2_@Ni material demonstrated higher hardness than that in the Cu-MoS_2_@Cu material. This difference is attributed to the solid solution strengthening effect of nickel, which forms an infinite solid solution with copper. In contrast, MoS_2_ reacts with copper during high-temperature sintering to form brittle phases [30]. Importantly, nickel does not react with MoS_2_ under the same conditions. This indicates that the incorporation of MoS_2_@Ni not only suppresses interfacial reactions but also increases the hardness of the Cu matrix and enhances the interfacial bonding strength.

**Figure 7:**
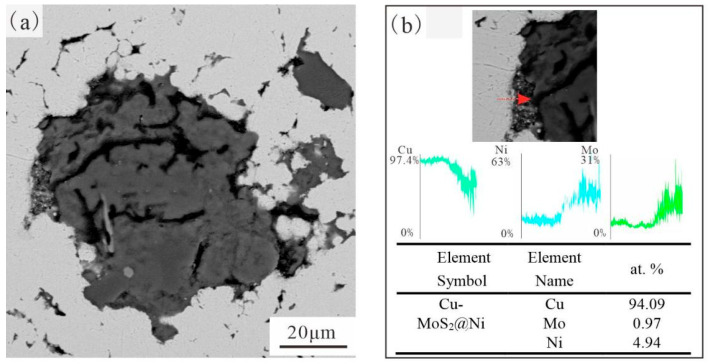
Analysis of the interface formed by MoS_2_@Cu and Cu matrix and the interface scan: (**a**) Cu-MoS_2_@Cu interface; (**b**) EDS analysis of selected area.

**Figure 8:**
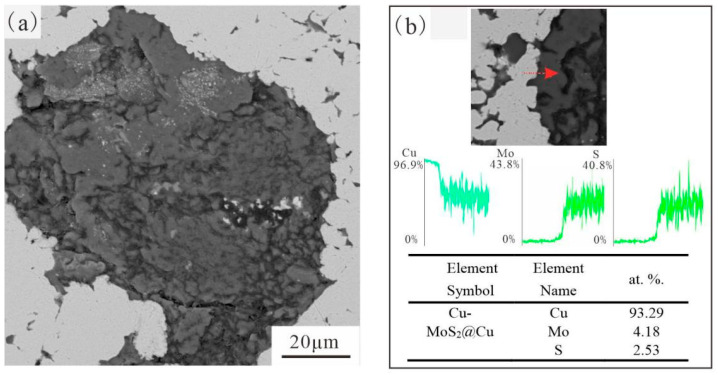
Morphology of the interface of MoS_2_@Ni and Cu matrix and EDS analysis of the interface: (**a**) interface of Cu-MoS_2_@Cu; (**b**) EDS analysis of the selected area.

Figure 7 and Figure 8 illustrate the detailed cross-section of Cu-MoS_2_ with different coatings and its interface with the matrix. The MoS_2_ with a Ni coating formed a tighter interfacial bond with the matrix. However, the MoS_2_@Cu seemed to have reacted with the matrix, led to the formation of some flocculent interface products. Further, EDS line scanning was employed to analyze the elemental changes at the interface between MoS_2_@Cu or MoS_2_@Ni with the matrix. For the MoS_2_@Ni/Cu interface, the concentrations of Cu and Ni elements at the interface shows a gradually increased tendency, indicating that there is a significant diffusion of metal elements at the interface. Although the MoS_2_@Cu/Cu interface also exhibits a subtle gradient change in interface metal elements, when scanning areas close to the interface reaction region, severe fluctuations in element concentration commence to occur. This very phenomenon proved the occurrence of the interface reaction. EDS analysis also indicated that the Ni coating contained P, suggesting that the deposited layer was a typical Ni–P alloy coating. The presence of P in the Ni–P coating may enhance hardness and corrosion resistance, which contributes to improved interfacial load-bearing capacity during micro-friction testing. In contrast, the Cu coating remains relatively soft and chemically active toward MoS_2_, facilitating interfacial reactions during sintering.

**Figure 9:**
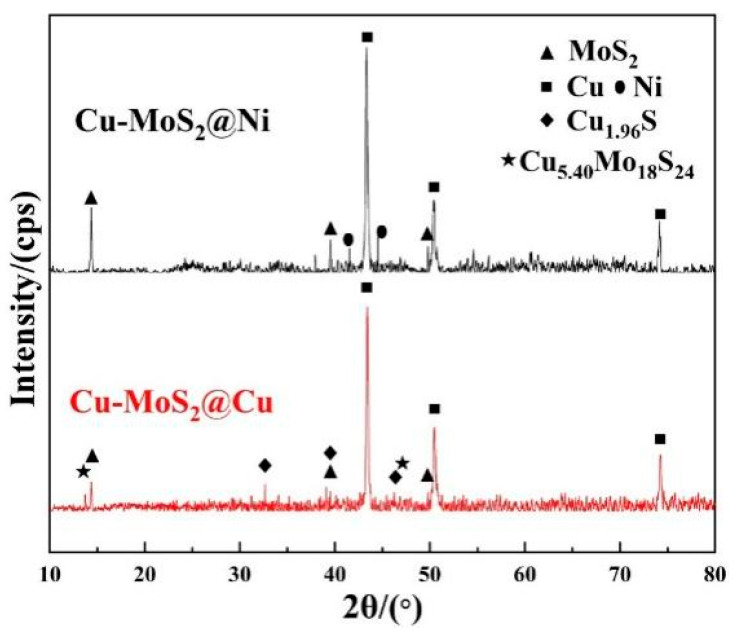
XRD diffraction patterns of Cu-based friction samples with different kinds of MoS_2_ after sintering.

To further explore the decomposition behavior of the two kinds of MoS_2_ after sintering, the phase composition of the Cu-MoS_2_@Cu and Cu-MoS_2_@Ni materials was characterized by XRD analysis, as shown in Figure 9. It is observed that after sintering, the sample containing MoS_2_@Cu exhibits a slight decomposition of MoS_2_, resulting in the formation of new sulfide phases, such as Cu1.96S and Cu5.40Mo18S24. In contrast, for the sample containing MoS_2_@Ni, the phase composition remains largely unchanged before and after sintering.

The results of EDS line analysis and XRD analysis indicate that, during the sintering process, MoS_2_@Cu exhibited a higher tendency towards decomposition. This phenomenon can be ascribed to a sequence of reactions occurring between MoS_2_ and Cu. The reaction equations are as follows:

(6) $$MoS_{2}+4Cu=2Cu_{2}S+Mo\;$$

(7) $$yMoS_{2}+2xCu=2Cu_{x}S_{y}+yMo\;$$

(8) $$\;\;yMoS_{2}+2xCu=2Cu_{x}Mo_{z}S_{y}+\left(y-2z\right)Mo\;$$

The △G of the reactions mentioned above are all consistently less than zero, indicating that these reactions took place spontaneously during the sintering process, with a sintering temperature of 870 °C; this was calculated using the Gibbs-free energy calculation software HSC Chemistry 6.0. This explains why multiple sulfides were detected in the XRD after sintering in the Cu-MoS_2_@Cu samples.

However, the reaction equation of MoS_2_ and Ni is as follows:

(9) $$yMoS_{2}+2xNi={2Ni}_{x}S_{y}+yMo\;$$

The reaction temperatures required for the above reaction equation all exceed 1100 °C (calculated using the Gibbs-free energy calculation software HSC Chemistry 6.0), which means that in this study, the Ni coating is capable of fulfilling a protective function, effectively inhibiting the significant reaction between MoS_2_ and the matrix. The analysis indicated the formation of two distinct interfacial structures with the matrix: MoS_2_@Ni exhibited a dense diffusion-bonded interface, as opposed to the defective diffusion-reaction bonded interface observed with MoS_2_@Cu.

### 3.2. Micro-Friction Tests

During actual braking, sliding friction occurs between the friction material and the counterpart. Since the Brinell hardness of the counterpart is significantly higher than that of the friction material, the friction process can be regarded as the slippage of microscopic asperities on the counterpart’s surface over the friction material’s surface. Therefore, investigating the micro-friction (micro-scratch) characteristics of the wear constituents and their interfaces with the matrix aims to provide an in-depth understanding of the friction performance and wear mechanisms exhibited by the friction material under real braking conditions. Understanding the micro-friction behavior helps explain the wear mechanisms of the composites and provides insight into the design of braking materials, improving their stability and durability under extreme conditions.

**Figure 10:**
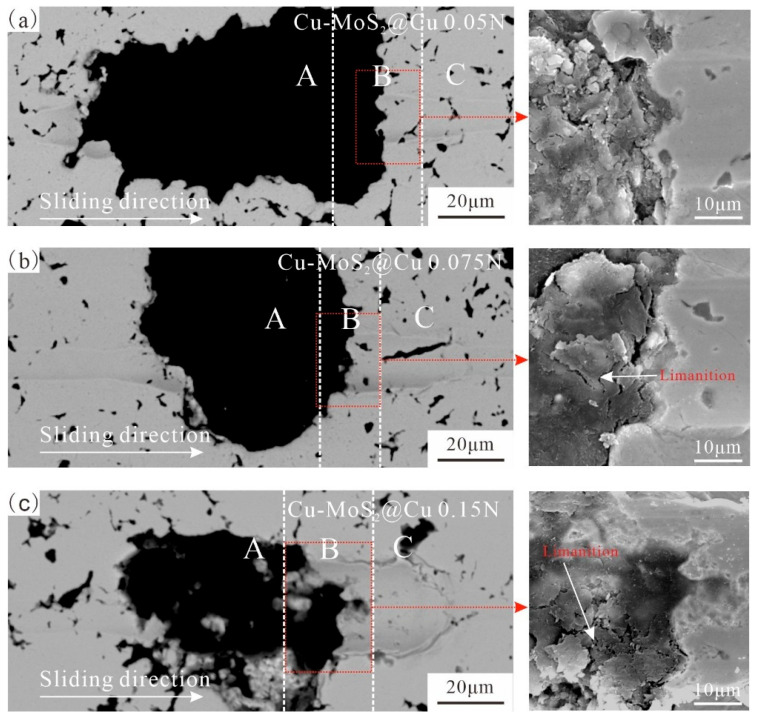
The SEM morphology of scratch on Cu-MoS_2_@Cu interface regions under different pressure conditions: (**a**) 0.05 N; (**b**) 0.075 N; (**c**) 0.15 N.

**Figure 11:**
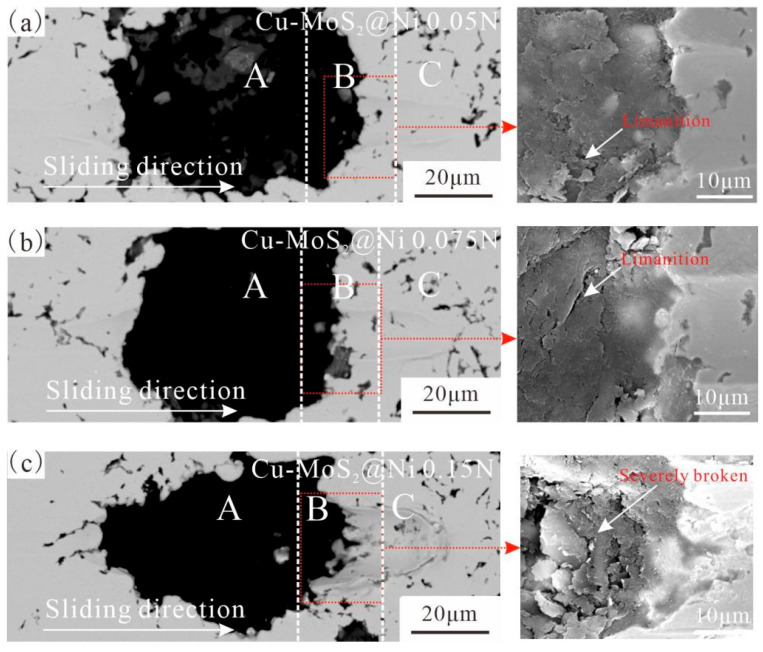
The SEM morphology of scratch on the Cu-MoS_2_@Ni interface regions under different pressures: (**a**) 0.05 N; (**b**) 0.075 N; (**c**) 0.15 N.

Figure 10 and Figure 11 show the scratch morphologies of the interfaces after micro-friction testing. In these figures, the black phase represents MoS_2_@Cu or Cu-MoS_2_@Ni, while the brighter phase corresponds to the Cu matrix. Because MoS_2_ appears dark in the SEM images, scratches on MoS_2_ are less visible than those on the matrix. An examination of micro-friction morphology on the interface and matrix reveals that as pressure increases, the width of the scratches significantly increases. While both samples exhibit generally similar scratch morphologies, notable differences are observed at the interfaces. However, the MoS_2_@Ni matrix interface demonstrates better resistance to damage under the same pressure conditions compared to the MoS_2_@Cu matrix interface.

Figure 10 and Figure 11 reveal the micro-frictional topography at the MoS_2_/matrix interfaces in greater detail in their right-hand sections. Under low applied pressure, the MoS_2_@Ni matrix interface remained intact, whereas the MoS_2_@Cu matrix interface showed signs of debonding and micro-cracking. Comparative analysis indicates that the load-bearing capacity of MoS_2_@Cu matrix interface is lower than that of MoS_2_@Ni. Specifically, interfacial delamination initiated near the MoS_2_@Cu interface at 0.05 N. Upon increasing the pressure to 0.075 N, the MoS_2_@Ni interface maintained its integrity, while the MoS_2_@Cu interface developed cracks and incipient failure, demonstrating the superior interfacial integrity of the former. As the pressure gradually increases to 0.15 N, MoS_2_ at the interface starts to show noticeable laminar slip, and a significant number of MoS_2_ flakes begin to form. Under this experimental condition, the delamination of MoS_2_ near the MoS_2_@Cu matrix became more pronounced. Based on the observed interfacial failure patterns, the MoS_2_@Cu interface exhibited significant interfacial debonding, whereas the MoS_2_@Ni interface maintained robust interfacial adhesion despite the fracture occurring within the MoS_2_.

The scratch characteristics of the interfaces between MoS_2_ and matrix exhibit a strong correlation with the structural properties of MoS_2_ and the performance of the interface between MoS_2_ and the Cu matrix. MoS_2_@Ni forms a stronger diffusion-bonded interface with the Cu matrix, leading to such interfaces being able to maintain better adhesion during the micro-friction tests. The strong interface of the MoS_2_@Ni matrix formed due to two main factors. First, at an 870 °C sintering temperature, Ni does not react with MoS_2_, delaying the MoS_2_-Cu reaction and reducing interfacial products. Second, the Ni-Cu diffusion rate is faster than Cu-Cu, promoting a high-strength diffusion bonding interface between the Ni coating and the matrix [39]. Thus, the Cu-MoS_2_@Ni interface shows no significant damage under various pressures. Nevertheless, MoS_2_@Cu formed a diffusion–reaction mixed bonding interface with the matrix. Due to the presence of interface defects, the bonding strength of this interface was relatively low. Consequently, interfacial debonding began to occur even under a test pressure of 0.075 N. Additionally, the relatively low strength of MoS_2_ and its layered structure render MoS_2_ highly susceptible to damage during micro-friction tests. This may even result in failure prior to the debonding of the interface. Therefore, during the micro-friction tests, the inability of MoS_2_ constituents and the interface debonding might occur simultaneously, collectively contributing to the interface failure. Notably, owing to the occurrence of interfacial reactions, the MoS_2_@Cu and MoS_2_@Cu matrix interface is more susceptible to damage in comparison with MoS_2_@Ni, leading to the damage of the interface even under the lowest pressure. Overall, with increasing pressure, the failure mechanism of the MoS_2_@Cu interface transitions from localized interfacial debonding to failure within the interfacial reaction layer and delamination of MoS_2_. For MoS_2_@Ni, the interfacial failure mechanism is characterized by delamination of MoS_2_ near the interface under high test pressure.

**Figure 12:**
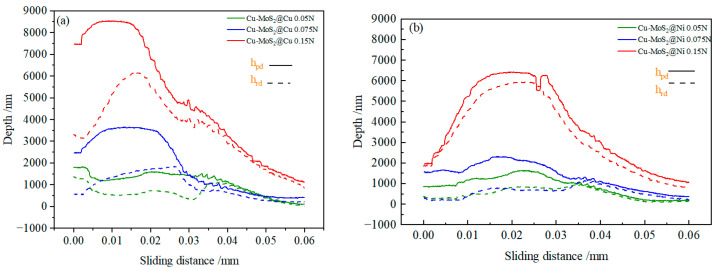
Variation curves of hrd and hpd in the Cu-MoS_2_@Cu and Cu-MoS_2_@Ni interface regions with the sliding distance under different regions: (**a**) Cu-MoS_2_@Cu interface; (**b**) Cu-MoS_2_@Ni interface.

Figure 12 displays the variation in residual depth (hrd) and penetration depth (hpd) with sliding distance for the interface regions A, B, and C in Cu-MoS_2_@Cu and Cu-MoS_2_@Ni samples under different pressure conditions. In micro-friction testing, both residual depth (hrd) and penetration depth (hpd) are critical indicators for evaluating hardness and deformation behavior (hpd refers to the maximum depth the indenter penetrates into the material’s surface under applied load, typically used to assess the material’s stiffness and hardness). Harder materials generally show smaller penetration depths. On the other hand, hrd represents the residual indentation depth remaining after unloading with elevated test pressures; however, the variation trend of the indentation depth exhibits distinct patterns depending on the specific characteristics of the test region. The permanent deformation depth left after the indenter is removed from the material’s surface during the unloading process reflects the extent of deformation, with softer materials typically leaving deeper residual depths. In the figures, A, B, and C represent three distinct test regions, referring to Cu-MoS_2_@Cu or the Cu-MoS_2_@Ni phase region, the interface region, and the matrix, respectively. Figure 12 presents the variations in hrd and hpd with sliding distance under different pressure conditions, as shown in Figure 10 and Figure 11. As illustrated, with an increasing sliding distance, the hrd and hpd show a discernible decreasing trend. In addition, the hrd and hpd consistently increase with elevated test pressures; however, the variation trend of the hrd and hpd exhibit distinct patterns depending on the specific characteristics of the test region. Owing to its relatively low hardness and propensity for interlayer delamination failure, the MoS_2_ region always exhibits a greater indentation depth and residual depth during mechanical testing. Under identical testing conditions, the indentation depth and residual depth in the MoS_2_@Cu phase exhibited relatively higher values. Particularly at a testing load of 0.15 N, the maximum indentation depth reached 8500 nm, and the residual depth reached 6300 nm. Conversely, the matrix region exhibits relatively lower indentation depth and residual depth due to its high hardness and toughness. The interfacial region, serving as a bridge connecting MoS_2_ and the substrate, exhibited a progressive reduction in both indentation depth and residual depth. The reduction in these depths became progressively smaller. Furthermore, the decrease in hpd and hrd at the MoS_2_@Ni/matrix interface was more gradual compared to that of MoS_2_@Cu.

In micro-scratch hardness testing, both hpd and hpd are associated with the material’s hardness and deformation characteristics, respectively. The indicator hpd refers to the scratch depth at which the indenter penetrates the material’s surface under applied loads, and is typically used to describe the actual insertion depth during the testing process. Harder materials generally show smaller penetration depths. On the other hand, hrd, representing the residual depth, is employed to reflect the extent of material deformation after the test. MoS_2_ exhibits relatively low hardness and is prone to tangential deformation during testing, resulting in increased hpd and hpd, whereas the relatively harder Cu-matrix shows a comparatively lower hpd and hrd.

The variation in indentation depth at the interface is associated with the load-bearing capacity of the interface. Generally speaking, the higher the interfacial strength between the two phases, the larger the load-bearing capacity of the interface. When the indenter slides across a high-strength interface, the change in indentation depth is more gradual. Conversely, a low-strength interface may be damaged as the indenter slides across, resulting in a sudden change in indentation depth due to the partial loss of load-bearing capacity. Therefore, micro-scratch tests, combined with post-experiment interfacial morphology characteristics, allow for a qualitative assessment of the interfacial performance between two phases. As shown in Figure 12, the indentation depth in interfacial region of the Cu-MoS_2_@Cu sample is higher compared to the Cu-MoS_2_@Ni interface. The change in indentation depth at the MoS_2_@Cu and Cu matrix interface initially fast increases and then decreases, indicating that the interface has been compromised under test pressures of 0.075 N and 0.15 N, leading to a deterioration in its load-bearing capacity. This result corresponds with the interfacial debonding phenomenon observed in Figure 10, clearly demonstrating the inferior mechanical performance of the Cu-MoS_2_@Cu matrix interface. This further confirms that the strength of the diffusion–reaction bonding interface in Cu-MoS_2_@Cu is lower than that of the diffusion bonding interface in Cu-MoS_2_@Ni.

**Figure 13:**
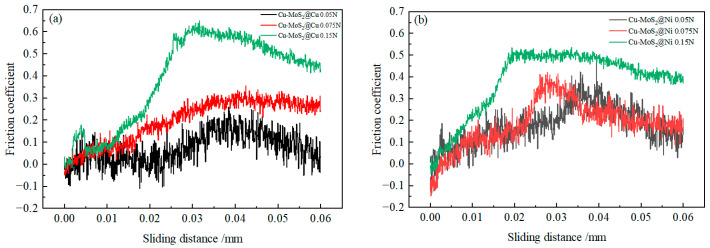
Variation curves of the friction coefficient in Cu-MoS_2_@Cu and Cu-MoS_2_@Ni interface regions with the sliding distance under different pressures: (**a**) Cu-MoS_2_@Cu interface regions; (**b**) Cu-MoS_2_@Ni interface regions.

Figure 13 presents the variation in coefficient of friction (COF) with sliding distance for the interfacial regions of Cu-MoS_2_@Cu and Cu-MoS_2_@Ni samples under different applied pressures. As illustrated, the coefficient of friction demonstrates an overall increasing trend with elevated pressure, exhibiting the most pronounced enhancement when the pressure reaches 0.15 N. A careful observation of the variation in the coefficient of friction with respect to sliding position reveals that the coefficient of friction remains relatively low when the indenter slides on the MoS_2_ phase. However, upon crossing the interface, the COF begins to increase, particularly when the applied test pressure reaches 0.15 N, at which a rapid escalation in the COF is observed. A comparative analysis of two distinct interface types indicates that under loading conditions of 0.05 N and 0.075 N, the friction coefficient at the interface maintains relative stability. Nevertheless, when the testing pressure is increased to 0.15 N, the MoS_2_@Cu/Cu interface shows a more prominent and abrupt change in the friction coefficient, with the maximum value possibly surpassing 0.6.

Based on the principles of adhesive friction theory [37], the coefficient of friction is given by:

(10) $$\;\;\;\mu =\mu_{a}+\mu_{p}\;$$

In the equation, μa represents an adhesive component of COF, and μp represents a plowing component of COF. The adhesive component during the sliding process can be calculated using the following equation [38]:

(11) $$\;\;\;\;\;\;\mu_{a}=\frac{\tau A_{v}}{{H_{v}A}_{v}}=\frac{\tau }{H_{v}}\;\;$$

In the equation, τ represents the material’s shear strength, and Hv denotes the material’s hardness. Equation (13) indicates that the adhesive friction component of the material during the testing process is only related to the intrinsic properties of the material and is independent of the testing conditions.

**Figure 14:**
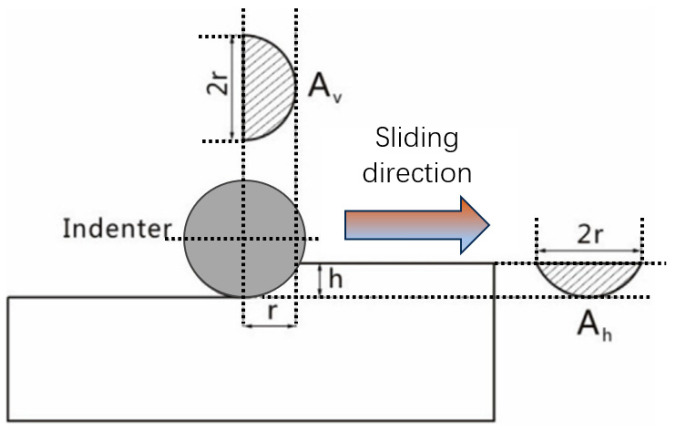
The schematic of indenter scratching on the surface.

A schematic of the micro-scratch test is presented in Figure 14. The plowing friction coefficient, $\mu_{p}$ which neglects the effect of elastic recovery on the contact contour, is given by:

(12) $$\mu_{p}=\frac{f_{p}}{N}=\frac{A_{h}\sigma_{h}}{A_{v}\sigma_{v}}\;$$

In this expression, $f_{p}$ is defined as the plowing component of the friction force. $A_{h}$ and $A_{v}$ are the horizontal and vertical projected areas of the contact zone, respectively, with the latter under plastic deformation. $\sigma_{h}$ and $\sigma_{v}$ correspond to the yield/compressive strength of the friction material along the sliding direction and the vertical direction, respectively.

For MoS_2_, its relatively low hardness results in a higher Ah/Av ratio. However, during the scratching process, the layered structure of MoS_2_ tends to shear and spread along the sliding direction, leading to extremely low values of both $\sigma_{h}$ and τ in that direction. With the escalation of test pressure, the enhancement of the $A_{h}$/$A_{v}$ ratio constitutes the main factors leading to growth in the COF. Material properties and structural characteristics of MoS_2_ facilitate the development of a low COF, thereby enabling superior lubrication performance.

For the friction calculation of the copper matrix, the matrix is a homogeneous material, and the yield stress in all directions is the same. Therefore, the ploughing component of the COF can be simplified as [21]

(13) $$\mu_{p}=\frac{A_{h}}{A_{v}}=\frac{R^{2}\cos^{-1}(\frac{R-h_{pd}}{R})-(R-h_{pd})\sqrt{2Rh_{pd}-{h_{pd}}^{2}}}{\frac{1}{2}\pi (2Rh_{pd}-{h_{pd}}^{2})}\;$$

In the equation, the R is the radius of indenter. Based on the correlation between indentation depth and hardness [9],

(14) $$h_{pd}=\frac{0.102F_{n}}{2\pi RH}\;$$

In the equation, Fn is the normal force. From Equations (13) and (14), it can be observed that the COF on the matrix is primarily governed by the variable plowing component. It is positively correlated with pressure and negatively correlated with hardness. The rapid increase in the COF from MoS_2_ to the matrix is attributed to two important factors: firstly, the enhancement of the adhesive component of COF due to the improved tangential strength of the surface; secondly, the swift rise in the $\sigma_{h}$/$\sigma_{v}$ ratio. Consequently, even when the indenter operated on the matrix, leading to a reduction in the $A_{h}$/$A_{v}$ ratio, the COF still exhibits a significant elevation.

The friction coefficient variation at the interface exhibits a more complex pattern. The variation in the COF correlates with the area distribution of the indenter’s contact with the material during the micro-friction tests, when the interface exhibits adequate load-bearing capacity and maintains structural integrity. The COF can be calculated as follows:

In the formula, α represents the coverage area of MoS_2_ on the contact surface. Under these circumstances, the variation in the COF should exhibit an approximately linear relationship with the sliding movement of the indenter. Under the operational conditions of 0.05 N and 0.075 N for both samples, the variation in the COF at the interface distinctly adheres to the pattern delineated by Equation (13). However, when the applied pressure was increased to 0.15 N, severe interfacial failure occurred at the Cu-MoS_2_@Cu interface, resulting in a rapid increase in indenter penetration depth. This led to an elevation in the Ah/Av ratio, causing a steep rise in the COF. Subsequently, as the indenter gradually slid onto the substrate, the friction coefficient began to decrease slowly. As a result, the COF at the Cu-MoS_2_@Cu interface formed a characteristic peak in the COF curve.

Due to its higher bonding strength, the MoS_2_@Ni-Cu matrix interface provides better support to the indenter during sliding. Consequently, its variations in indentation depth and COF were more stable compared to those of the MoS_2_@Cu-Cu matrix interface. The higher bonding strength of the MoS_2_@Ni-Cu matrix interface compared to the MoS_2_@Cu-Cu matrix interface, as evidenced by the COF variations indicative of interfacial failure in micro-scratch testing, accounts for its more stable tribological behavior.

### 3.3. Macroscopic Frictional Behavior of the Cu-Based Composites

Macro-friction tests were conducted on a scaled-down friction tester to investigate the tribological behavior of Cu-based composites containing two modified lubricating components under different braking energy densities. This research explores the characteristics of the modified lubricating components, their interface features with the matrix, and their relationship to friction and wear performance.

Table 4 presents the theoretical density, measured density, and relative density values of Cu-BC-MoS_2_@Cu and Cu-BC-MoS_2_@Ni materials. As shown in the table, the measured density of Cu-BC-MoS_2_@Ni (7.92 g/cm^3^) is higher than that of Cu-BC-MoS_2_@Cu (7.88 g/cm^3^), with both materials exhibiting similar densities. It can be found that, during the sintering process, the reaction between MoS_2_ and Cu is more extensive in Cu-BC-MoS_2_@Cu than in Cu-BC-MoS_2_@Ni, resulting in the formation of more brittle phases. Additionally, Cu and Ni form an infinitely soluble solid solution with fewer internal defects, thus slightly increasing the density of the Cu-BC-MoS_2_@Ni material.

Porosity plays a critical role in determining the tribological behavior of composite materials. As presented in Table 4, both composites exhibit relative densities of approximately 91–92%, corresponding to a porosity level of about 8–9%. Notably, Cu BC MoS_2_@Ni demonstrates a slightly higher density (7.92 g/cm^3^ vs. 7.88 g/cm^3^) and thus marginally lower porosity compared to its counterpart. The presence of porosity exerts a dual influence on tribological performance. On one hand, pores can act as stress concentrators, potentially reducing local mechanical strength and facilitating crack initiation under cyclic loading, particularly under high-energy-density conditions. On the other hand, pores may serve as reservoirs for wear debris and contribute to the mechanical interlocking of tribolayers, which can, in some cases, enhance the adhesion of oxide films. In the present study, for Cu BC MoS_2_@Cu, which possesses a weaker interfacial bonding and higher porosity, the synergistic effect of these factors exacerbates subsurface crack propagation, thereby promoting the occurrence of delamination wear at an energy density of 100 J/mm^2^. In contrast, the denser matrix (attributed to slightly lower porosity) and stronger interfacial bonding of Cu BC MoS_2_@Ni effectively inhibit crack growth, confining wear predominantly to the oxide layer rather than extending into the bulk material. Furthermore, the marginally reduced porosity in Cu BC MoS_2_@Ni contributes to its higher microhardness (Figure 6) and enhanced resistance to plastic deformation during sliding, which aligns well with its more stable friction coefficient and lower wear rate.

**Figure 15:**
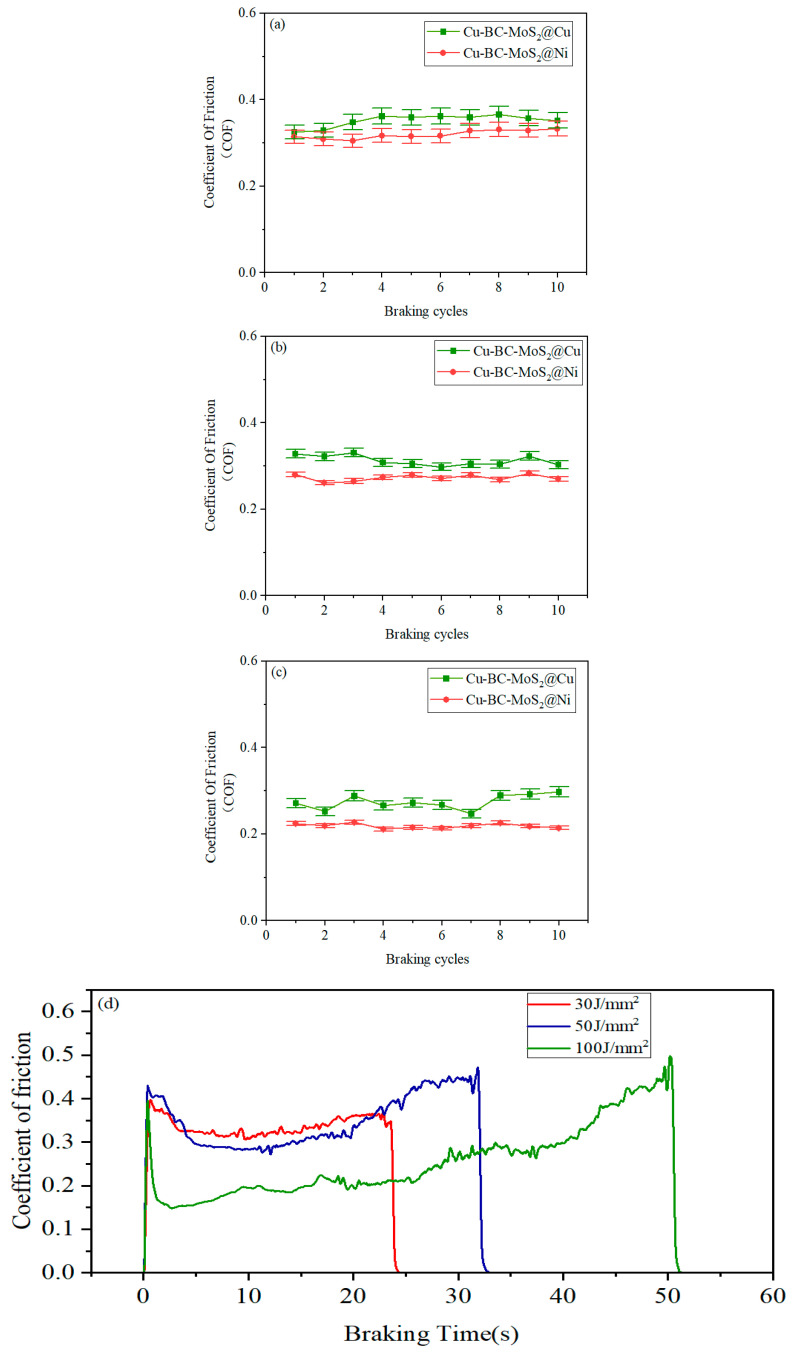

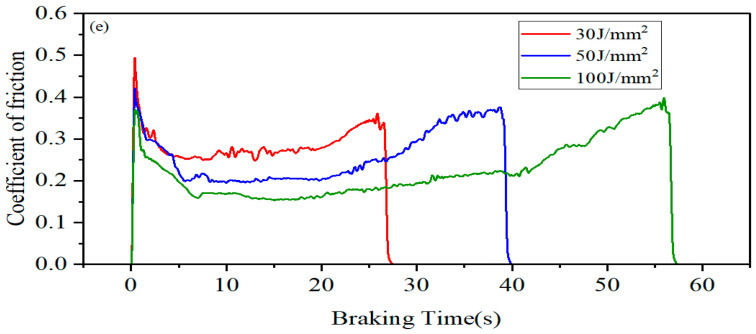
Variation curve of friction coefficient of Cu-BC-MoS_2_@Cu and Cu-BC-MoS_2_@Ni materials with number of test and Cu-BC-MoS_2_@Cu/Ni materials at different braking energy densities: (**a**) Cu-BC-MoS_2_@Cu/Ni 30 J/mm^2^; (**b**) Cu-BC-MoS_2_@Cu/Ni 50 J/mm^2^ (**c**) Cu-BC-MoS_2_@Cu/Ni 100 J/mm^2^; (**d**) Cu-BC-MoS_2_@Cu instantaneous friction coefficients; (**e**) Cu-BC-MoS_2_@Ni instantaneous friction coefficients.

**Figure 16:**
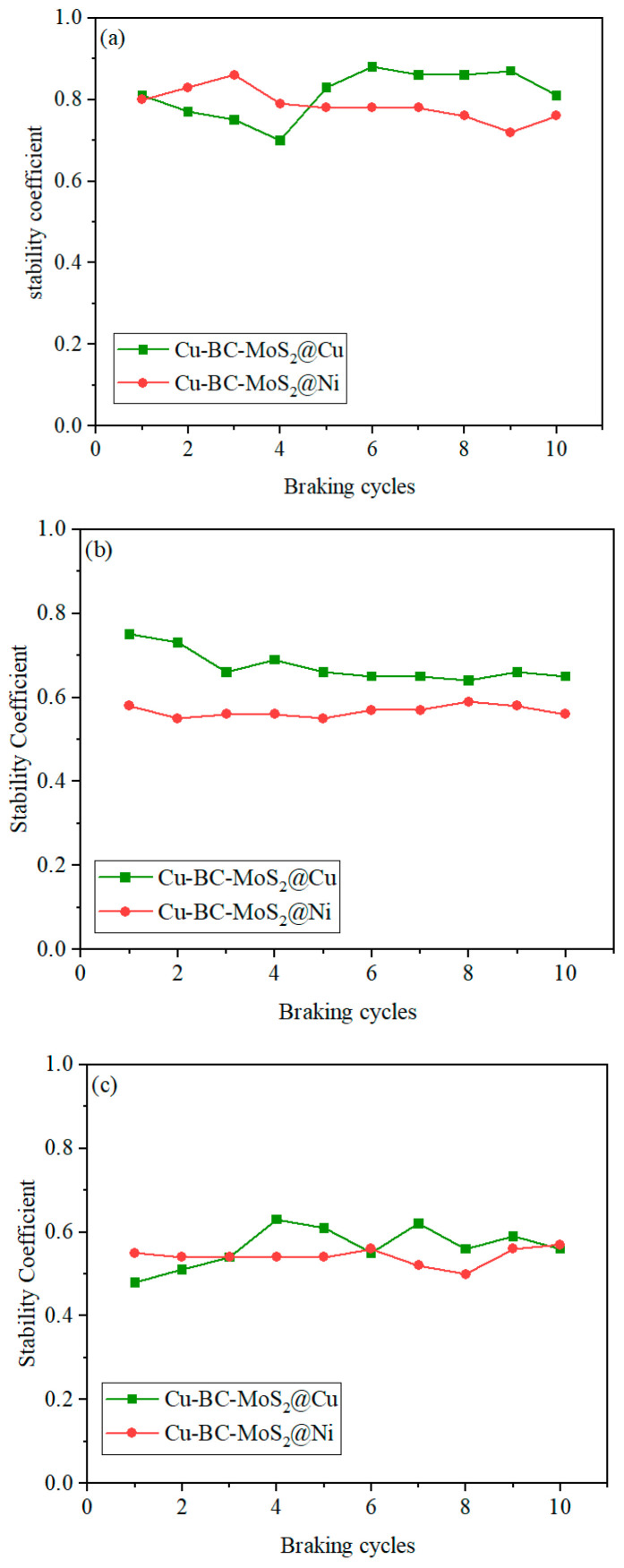
The stability coefficient of Cu-BC-MoS_2_@Cu and Cu-BC-MoS_2_@Ni at different energy densities: (**a**) 30 J/mm^2^; (**b**) 50 J/mm^2^; (**c**) 100 J/mm^2^.

Figure 15 shows the variation in the friction coefficients of Cu-BC-MoS_2_@Cu and MoS_2_@Ni materials with the number of braking cycles. As shown in Figure 16, under the same velocity conditions, both materials exhibit high stability in their friction coefficients. However, MoS_2_@Ni demonstrates a more stable friction coefficient compared to MoS_2_@Cu. Furthermore, with an increase in speed, the friction coefficients of both materials tend to decrease, with MoS_2_@Ni maintaining good stability.

Figure 15d,e show that the friction curves of both materials generally exhibit a saddle-shaped profile. As the energy density increases, the curvature on both sides of the friction curve becomes more pronounced. The appearance of this saddle-shaped friction curve is primarily related to the contact state during the friction process. During the initial stage of the friction test, the impact generated by the contact of the friction pair is the main factor leading to the initial peak in the braking curve. In the middle stage, the formation of a friction film causes the instantaneous COF to gradually decrease and stabilize. At the final stage, due to the decreasing rotational speed and intensified heat accumulation at the friction interface, the interlocking engagement between the friction material and the counterpart is enhanced, resulting in a rapid increase in the COF. This manifests as a more pronounced “tail-up” phenomenon in the friction termination stage with increasing friction speed.

The slightly lower friction coefficient of Cu-BC-MoS_2_@Ni compared to Cu-BC-MoS_2_@Cu can be attributed to the formation of a weaker reaction–diffusion interface between MoS_2_@Ni and the copper substrate. In contrast, MoS_2_@Cu reacts with the substrate to form a stronger reaction interface, which, although having lower strength, is more easily damaged during friction. This results in the generation of fine abrasive particles that contribute to third-body wear, thereby enhancing friction. MoS_2_ has a hexagonal close-packed crystal structure, with strong in-plane atomic bonding and relatively weak interlayer bonding. During friction, the interlayer chemical bonds of MoS_2_ break, forming a lubricating sulfide film that provides excellent lubrication performance. Furthermore, the interface between MoS_2_@Ni and the substrate generates fewer sulfides and hard phases, preserving more MoS_2_, thereby enhancing its lubricating effect.

As shown in Figure 15 and Figure 16, both Cu-BC-MoS_2_@Cu and Cu-BC-MoS_2_@Ni exhibit good stability coefficients under low-energy-density conditions. As the energy density increases, the higher hardness of Cu-BC-MoS_2_@Ni leads to reduced material stability during friction. However, the continuous lubrication effect of MoS_2_ reduces the tendency for adhesion, allowing Cu-BC-MoS_2_@Ni to maintain stability at high speeds, with minimal change in stability compared to Cu-BC-MoS_2_@Cu. Furthermore, the stronger interfacial bonding between Cu and Ni enhances the material’s resistance to deformation, thereby reducing frictional resistance and lowering the friction coefficient. Furthermore, the high heat capacity of molybdenum (Mo) during its decomposition enables it to absorb a significant amount of frictional heat, lowering the surface temperature and thereby further promoting the stability of the COF.

**Figure 17:**
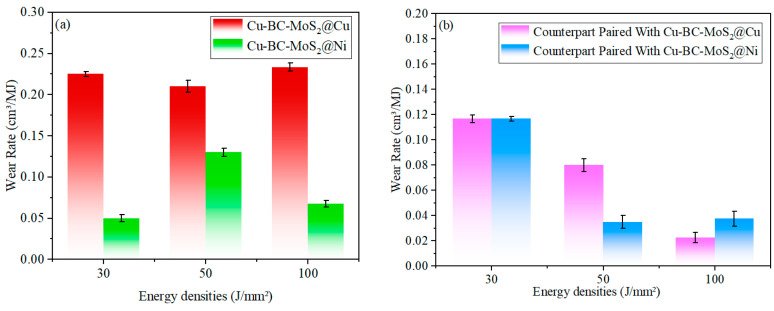
Wear loss of Cu-BC-MoS_2_@Cu and Cu-BC-Cu-BC-MoS_2_@Ni materials and their counterparts under different energy densities: (**a**) Cu-BC-MoS_2_@Cu and Cu-Cu-BC-MoS_2_@Ni; (**b**) counterpart.

**Figure 18:**
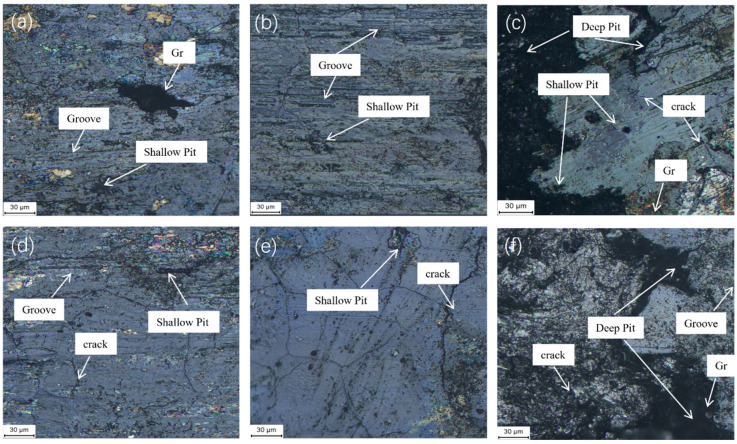
Macroscopic morphology of friction surface of Cu-BC-MoS_2_@Cu and Cu-BC-MoS_2_@Ni materials with different energy densities: (**a**) Cu-BC-MoS_2_@Cu 30 J/mm^2^; (**b**) Cu-BC-MoS_2_@Cu 50 J/mm^2^; (**c**) Cu-BC-MoS_2_@Cu 100 J/mm^2^; (**d**) Cu-BC-MoS_2_@Ni 30 J/mm^2^; (**e**) Cu-BC-MoS_2_@Ni 50 J/mm^2^; (**f**) Cu-BC-MoS_2_@Ni 100 J/mm^2^.

Figure 17 presents the wear rates of Cu-BC-MoS_2_@Cu and Cu-BC-MoS_2_@Ni materials, along with their comparative materials, under different energy densities. Figure 18 illustrates the evolution of the friction surface and the transition of the wear mechanism of Cu-BC-MoS_2_@Cu and Cu-BC-MoS_2_@Ni composite materials based on copper, at energy densities of 30, 50, and 100 J/mm^2^.

At a low energy density of 30 J/mm^2^, the accumulation of frictional heat is restricted, thereby preventing the formation of a stable lubricating film on the surface. Friction primarily occurs through the mechanical action of micro-protrusions. For Cu-BC-MoS_2_@Cu, the weak bonding at the Cu-MoS_2_ interface leads to early consumption of MoS_2_ through reaction with the copper substrate. This results in the poor retention of the lubricating phase and prevents the formation of an effective lubricating film. This leads to the detachment of hard-phase particles, such as CuMoS_2_, contributing to third-body wear, during friction. The surface exhibits typical plowing wear morphology, with numerous plowing grooves and pits, and even exposes the Cu substrate. In contrast, Cu-BC-MoS_2_@Ni forms solid solutions with the Cu substrate, producing solid-solution strengthening and a stronger interface bond. This retains more MoS_2_ and allows for the formation of an effective lubricating film. As a result, the surface wear is lighter, primarily showing plowing wear with only minor adhesive phenomena.

As the energy density increases to 50 J/mm^2^, the interface temperature and contact pressure rise significantly, leading to surface oxidation and the formation of a light gray oxide film. For Cu-BC-MoS_2_@Cu, the increase in oxide film hardness reduces the number of plowing grooves. However, due to the relatively weak bonding at the Cu-MoS_2_ reaction interface, the oxide film is prone to detachment from the substrate, resulting in flaky spalling pits. This causes the wear mechanism to transition from purely plowing wear to a composite mechanism, including plowing, adhesion, and minor oxidative wear. On the other hand, due to the high-strength diffusion bond interface between Ni and MoS_2_, Cu-BC-MoS_2_@Ni forms a more compact oxide film, which is less prone to spalling. The surface integrity is better, and the overall wear rate is lower compared to Cu-BC-MoS_2_@Cu.

At medium energy density, although Cu-BC-MoS_2_@Ni forms a more compact oxide film than at low energy density, the film formation rate is slower than its consumption rate during friction. Therefore, at this medium energy density, the wear rate of Cu-BC-MoS_2_@Ni is the highest. For Cu-BC-MoS_2_@Cu, the oxide film can form under medium-energy-density friction, whereas Cu-BC-MoS_2_@Cu cannot generate an oxide film under low energy density and lacks the lubrication from residual MoS_2_ during the friction process. As a result, the wear rate of Cu-BC-MoS_2_@Cu is the lowest at medium energy density.

When the energy density is further increased to 100 J/mm^2^, the accumulation of frictional heat intensifies, and both materials’ surfaces become covered with a continuous, smooth oxide film. The number of spalling pits increases significantly, and deep pits begin to appear. On Cu-BC-MoS_2_@Ni, the oxide film is firmly bonded to the substrate, forming a dense oxide layer with localized high-temperature oxidation zones, indicating a more complete oxidation reaction. This further enhances the surface hardness and wear resistance. At this stage, although the wear mechanism primarily involves oxide film fatigue delamination, the degree of spalling is lighter, and the wear rate is lower than that observed at medium energy densities.

For Cu-BC-MoS_2_@Cu, due to insufficient interface bonding strength, the oxide film undergoes fatigue delamination under cyclic shear stress, with large-scale flaky spalling becoming the predominant wear mechanism. This results in a rapid increase in the wear rate, which reaches its highest value across all three energy densities. In contrast, the oxide film on Cu-BC-MoS_2_@Ni is strongly bonded to the substrate, featuring a dense oxide layer and localized high-temperature oxidation zones, which suggest a more complete oxidation reaction. These factors contribute to an enhancement in surface hardness and wear resistance. Although the wear mechanism still primarily involves fatigue delamination of the oxide film, the extent of spalling is less severe, and the wear rate is lower than that observed at medium energy densities.

**Figure 19:**
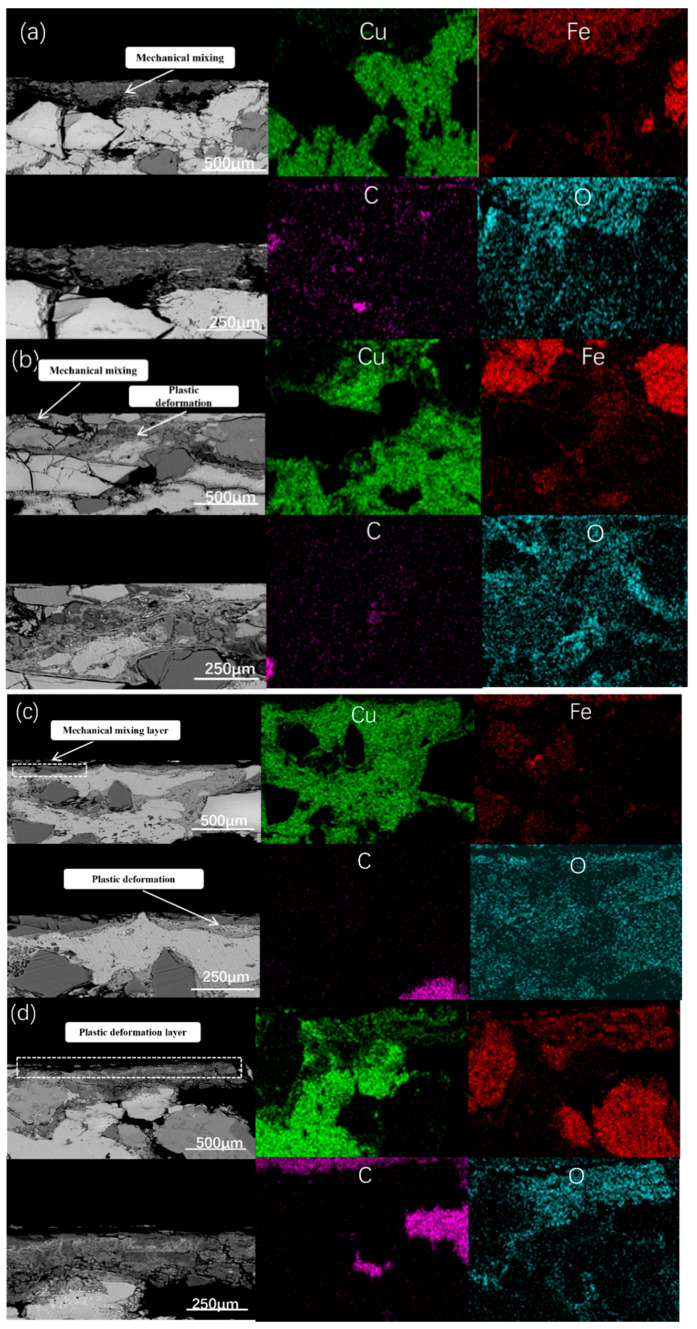

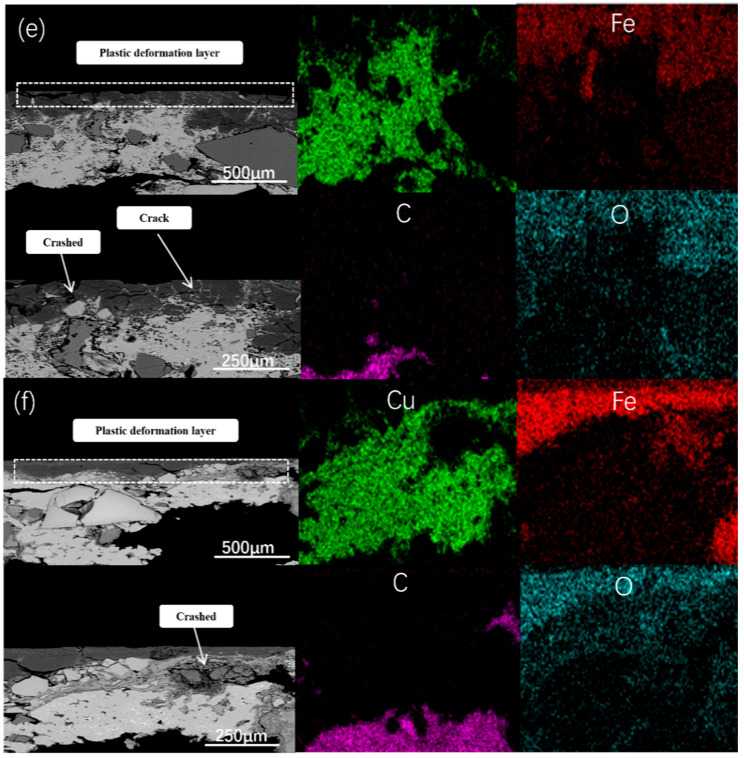
SEM and EDS images of sub-surface of friction materials under different energy densities (BSE): (**a**) Cu-BC-MoS_2_@Cu 30 J/mm^2^; (**b**) Cu-BC-MoS_2_@Cu 50 J/mm^2^; (**c**) Cu-BC-MoS_2_@Cu 100 J/mm^2^; (**d**) Cu-BC-MoS_2_@Ni 30 J/mm^2^; (**e**) Cu-BC-MoS_2_@Ni 50 J/mm^2^; (**f**) Cu-BC-MoS_2_@Ni 100 J/mm^2^.

Figure 19 illustrates the evolution of the cross-sectional structure under varying braking energy density (BED) conditions. As the rotational speed increases, distinct subsurface structures gradually emerge.

As shown in Figure 19a,b, at low BED, neither Cu-BC-MoS_2_@Cu nor Cu-BC-MoS_2_@Ni exhibit a well-defined subsurface structure. Instead, only discontinuous mechanical mixing and plastic deformation are observed. When the BED increases to 50 J/mm^2^, although neither of the two composite materials forms a continuous subsurface structure, Cu-BC-MoS_2_@Cu shows a discontinuous mechanical mixing layer with minor plastic deformation, as depicted in Figure 19c. In contrast, Cu-BC-MoS_2_@Ni maintains superior integrity after testing, exhibiting some discontinuous plastic deformation layers.

As the BED increases further, a mechanical mixing layer approximately 20 µm thick, enriched with iron, forms on the wear surface of the Cu-BC-MoS_2_@Cu sample. This layer is filled with cracks. However, as shown in Figure 19f, on the wear surface of the Cu-BC-MoS_2_@Ni, a plastic deformation layer of uneven thickness but continuous structure forms. Additionally, incomplete mechanical mixing layers are observed in some regions of this layer. This progression highlights the gradual development of subsurface structures as the BED increases, with Cu-BC-MoS_2_@Ni demonstrating better structural stability compared to Cu-BC-MoS_2_@Cu under higher energy conditions.

In conclusion, the interface bonding strength and the stability of the oxide film are critical factors determining the wear behavior of these materials at different energy densities. At low energy densities, interface bonding strength governs the retention of the lubricating phase; at medium energy densities, interface bonding influences the spalling tendency of the oxide film; and at high energy densities, interface bonding strength dictates the fatigue life of the oxide film and the overall wear resistance.

**Figure 20:**
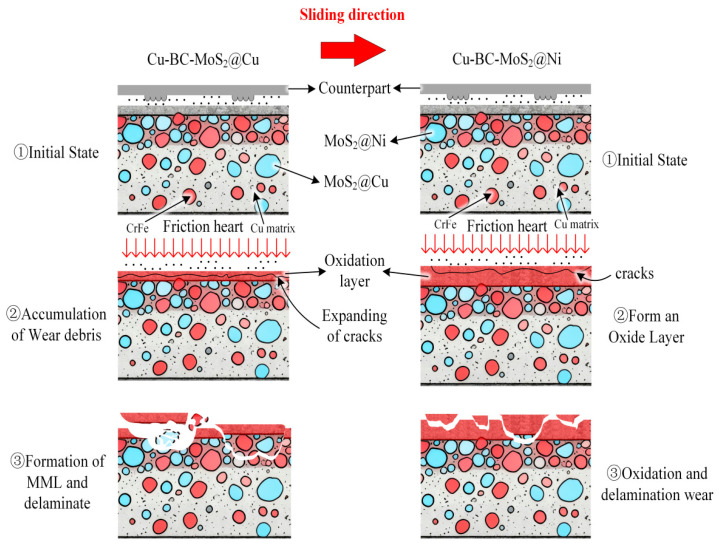
Wear schematic of the Cu-BC-MoS_2_@Cu and Cu-BC-MoS_2_@Ni materials.

As shown in Figure 20, the analysis of the friction surfaces indicates that for both Cu-BC-MoS_2_@Cu and Cu-BC-MoS_2_@Ni materials, under low-energy-density conditions, due to the incomplete formation of MoS_2_-containing oxide films, a large number of plow grooves and adhesion pits appear due to third-body wear caused by uneven surface protrusions. Under these conditions, both materials primarily experienced abrasive and adhesive wear. With increasing braking energy densities, the progressive formation of a surface oxide layer and debris coverage occurred. The thicker oxide film developed on Cu-BC-MoS_2_@Ni, compared to Cu-BC-MoS_2_@Cu, provided more effective protection against matrix wear and fracture. During high-energy braking, the formation rate of the oxide film is lower than its consumption rate, and the substances formed by the reaction between MoS_2_ and Cu increase the brittleness of the material. The weaker interfacial bonding makes the material more prone to damage during friction, leading to a shift in the primary wear mechanism of Cu-BC-MoS_2_@Cu to fatigue delamination of the material. In contrast, Cu-BC-MoS_2_@Ni, with its thicker oxide film, primarily experiences fatigue delamination of the oxide film.

## 4. Conclusions

This study systematically investigated the microstructure characteristics of two types of coated MoS_2_ materials, their interfacial bonding performance with the matrix, and their tribological behavior at both micro- and macro-scales within copper-based composites. The mechanisms by which these two coatings influence the tribological properties of the composites were elucidated. Furthermore, the evolution of friction and wear mechanisms in materials containing these differently coated MoS_2_ particles under varying relative sliding and rotational energy densities was unveiled. The main conclusions drawn from this research are as follows:

(1) During the sintering process, the MoS_2_@Cu composite undergoes an interfacial reaction between MoS_2_ and the Cu matrix, resulting in the decomposition of MoS_2_ and the formation of a diffusion-bonded interface accompanied by interfacial defects. In contrast, the MoS_2_@Ni composite develops a robust diffusion-bonded interface with the matrix, demonstrating enhanced interfacial strength and stability.
(2) The MoS_2_@Ni composite preserves a greater amount of MoS_2_ due to the lack of chemical interaction with the nickel matrix, thereby exhibiting improved lubricating characteristics, as indicated by a lower and more stable COF. Conversely, the lower hardness of MoS_2_@Cu makes it susceptible to fragmentation during micro-sliding, particularly under a 1.5 N load, resulting in an elevated and more unstable friction coefficient.
(3) Weaker interfacial bonding in MoS_2_@Cu leads to accelerated wear under high energy density conditions. The weak interfacial bonding promotes fatigue-induced delamination of the oxide layer during sliding. In comparison, MoS_2_@Ni features stronger interfacial cohesion, which enhances oxide layer adhesion to the matrix, thereby reducing delamination and contributing to more stable wear performance.
(4) In macro-scale tests, Cu-BC-MoS_2_@Ni exhibits a substantially lower wear rate than Cu-BC-MoS_2_@Cu—particularly under high energy densities, where reductions exceed 30%. Furthermore, Cu-BC-MoS_2_@Ni demonstrates more consistent frictional behavior and milder surface degradation, underscoring its superior wear resistance under high-load and high-speed sliding conditions.
(5) At low energy densities, both Cu-BC-MoS_2_@Cu and Cu-BC-MoS_2_@Ni experience abrasive wear as the prevailing mechanism. With increasing energy input, the wear mechanism transitions to a combination of abrasive and adhesive wear. Under high energy densities, Cu-BC-MoS_2_@Cu is predominantly characterized by fatigue delamination wear, leading to elevated wear rates. In contrast, Cu-BC-MoS_2_@Ni undergoes delamination wear within the oxide layer, which maintains oxide film integrity and results in lower wear rates, thereby affirming its improved wear resistance.

## Figures and Tables

**Table 1:** The main characteristic parameters of the raw material powder.

Raw Material	Component Content	Manufacturer	Particle Size
Copper	Cu ≥ 99.98%, O ≤ 0.01%	Youyan Company, Beijing, China	≤75 μm
Gr	Graphite ≥ 97%	Qingdao Shengtai Graphite Co., Ltd., Qingdao, China	≤300 μm
CrFe	Cr ≥ 53%, C ≤ 0.08%,		
P ≤ 0.04%, Fe balance	Zhongxin Manganese Industry Company, Ningxia, China	≤75 μm	
Fe	Fe ≥ 99.8%	Wugang Company, Wuhan, China	≤100 μm
MoS_2_@Cu	MoS_2_ ≥ 70%	Shanghai Huayi Group Corporation Limited, Shanghai, China	≤100 μm

**Table 2:** Constituents of the Cu-based composites (vol. %).

Element	Cu	ZrO_2_	Graphite	MoS_2_@Cu	MoS_2_@Ni	Others
1#	60	/	/	40		/
2#	60	/	/		40	/
3#	45~55	3~7	15~20	10	/	5–10
4#	45~55	3~7	15~20	/	10	5–10

**Table 3:** Parameters of interface micro-friction experiment.

Sample	Load	Sliding Distance	Sliding Speed	Relative Humidity
Cu-MoS_2_@Cu	0.05 N/0.075 N/0.15 N	60 μm	0.2 mm/min	50%
Cu-MoS_2_@Ni	0.05 N/0.075 N/0.15 N	60 μm	0.2 mm/min	50%

**Table 4:** Theoretical density, measured density and relative density of Cu-BC-MoS_2_@Cu and Cu-BC-MoS_2_@Ni materials.

Sample	Theoretical Density (g/cm^3^)	Measured Density (g/cm^3^)	Relative Density (%)
Cu-BC-MoS_2_@Cu	8.64	7.88	91.2
Cu-BC-MoS_2_@Ni	8.64	7.92	91.7

## Data Availability

The original contributions presented in this study are included in the article. Further inquiries can be directed to the corresponding authors.
